# Use of Dichloramin-T

**Published:** 1919-01

**Authors:** 


***Use of Dichloramin-T.*** By W. E. Lee and W. H. Turner, Philadelphia, *Journal A. M. A.*, Sept. 14, 1918.

The authors say that the experience of American surgeons in this war agrees with that of other surgeons of France and England; namely, that chlorin preparations have proved superior to all other germicides in military and other surgery. According to Dakin's views the germicidal action of all chlorin preparations depends on substances resulting from the chemical reactions between the chlorin and the proteins of the tissues and exudates in infected wounds, which substances are known as chloramins. These chloramins, unlike chlorin, are not destructive to the tissues, and may be used in solutions as strong as 10 per cent., and, therefore, it is not necessary to use the very weak concentrations, which require frequent applications, most essential when chlorin solutions are used. One of the authors refers to the commission of which he was a member, appointed in June, 1917, at the Boston meeting of the American Surgical Association to investigate the clinical value of dichloramin-T, and quotes the concluding paragraph of their first report stating the qualities of a proper germicide. These are given by him now in more detail. The irritation that accompanies the application of most germicides, limiting their use and governing their permissible concentrations, is negligible with dichloramin-T.

After fifteen months' work the authors have arrived at the following conclusions: " 1. The use of dichloramin-T has definitely improved the results we have been able to obtain in the primary closure of traumatic wounds of the soft tissues, bones and joints. 2. In the treatment of superficial accessible surgical infections the use of dichloramin-T has uniformly given us better results than any other germicide we have employed, and the method of its application is simpler and the dressings are more economical than with any of the other chlorin agents. 3. The best results with dichloramin-T can be obtained only when actual chemical contact of the germicide with the infecting organism is maintained. To maintain such contact in superficial surgical infections is a simple matter, and in the first few months of the work a satisfactory technic for this class of wounds was developed. In deep and inaccessible infections the problem is more difficult, and the greater part of these fifteen months has been devoted to this aspect. 4. Our confidence in the germicidal value of dichloramin-T has so developed that when it does not control a surgical infection we believe that the chemical contact has not been maintained, the mass of the germicide employed has not been sufficient, or adequate surgical treatment has not been given. 5. The striking detoxicating effect of the chlorin group of germicides, which has become common knowledge through the general use of Dakin's hypochlorite solution, is just as satisfactorily exhibited with dichloramin-T."

